# Alkaline phosphatase-to-albumin ratio as a novel predictor of long-term adverse outcomes in coronary artery disease patients who underwent PCI

**DOI:** 10.1042/BSR20203904

**Published:** 2021-06-29

**Authors:** Xin-Ya Dai, Ying-Ying Zheng, Jun-Nan Tang, Wei Wang, Qian-Qian Guo, Shan-Shan Yin, Jian-Chao Zhang, Meng-Die Cheng, Feng-Hua Song, Zhi-Yu Liu, Kai Wang, Li-Zhu Jiang, Lei Fan, Xiao-Ting Yue, Yan Bai, Zeng-Lei Zhang, Ru-Jie Zheng, Jin-Ying Zhang

**Affiliations:** 1 Department of Cardiology, First Affiliated Hospital of Zhengzhou University, Zhengzhou 450052, China; 2 Key Laboratory of Cardiac Injury and Repair of Henan Province, Zhengzhou, China; 3 Henan Medical Association, Intersection of Jinshui East Road and Boxue Road, Jinshui District, Zhengzhou 450000, China; 4 Henan Academy of Medical Sciences, Intersection of Jinshui East Road and Boxue Road, Jinshui District, Zhengzhou 450003, China

**Keywords:** Alkaline phosphatase-to-albumin ratio, Coronary artery disease, Long-term adverse outcomes, Percutaneous coronary intervention

## Abstract

**Background:** Alkaline phosphatase (ALP) and albumin (ALB) have been shown to be associated with coronary artery disease (CAD), and it has been reported that alkaline phosphatase-to-albumin ratio (AAR) is associated with the liver damage and poorer prognosis of patients with digestive system malignancy. Moreover, several previous studies showed that there was a higher incidence of malignancy in CAD patients. However, to our knowledge, the relationship between AAR and long-term adverse outcomes in CAD patients after undergoing percutaneous coronary intervention (PCI) has not been investigated. Therefore, we aim to access the relation between AAR and long-term adverse outcomes in post-PCI patients with CAD.

**Methods:** A total of 3378 post-PCI patients with CAD were enrolled in the retrospective Clinical Outcomes and Risk Factors of Patients with Coronary Heart Disease after PCI (CORFCHD-ZZ) study from January 2013 to December 2017. The median duration of follow-up was 37.59 ± 22.24 months. The primary end point was long-term mortality including all-cause mortality (ACM) and cardiac mortality (CM). The secondary end points were major adverse cardiac events (MACEs) and major adverse cardiac and cerebrovascular events (MACCEs).

**Results:** Kaplan–Meier analyses showed that an increased AAR was positively correlated with incidences of long-term ACM (log-rank, *P*=0.014), CM (log-rank, *P=*0.011), MACEs (log-rank, *P=*0.013) and MACCEs (log-rank, *P=*0.006). Multivariate Cox regression analyses showed that the elevated AAR was an independent predictor of long-term ACM (adjusted HR = 1.488 [1.031–2.149], *P*=0.034), CM (adjusted HR = 1.837 [1.141–2.959], *P=*0.012), MACEs (adjusted HR = 1.257 [1.018–1.551], *P*=0.033) and MACCEs (adjusted HR = 1.237 [1.029–1.486], *P*=0.024).

**Conclusion:** An elevated AAR is a novel independent predictor of long-term adverse outcomes in CAD patients following PCI.

## Introduction

Coronary artery disease (CAD) has been a major cause of mortality, and researches on CAD have drawn intense attention worldwide [1]. Previous studies demonstrated that several biomarkers were significantly associated with the pathogenesis of CAD: inflammatory response such as high-sensitivity C-reactive protein (hsCRP) [2] and interleukin-6 (IL-6) [3]; lipid metabolism such as triglyceride-rich lipoprotein-cholesterol (TRL-C) [4] and low-density lipoprotein cholesterol (LDL-C) [5]; hypercoagulability such as d-dimer [6] and fibrin degradation products (FDPs) [7]; atherosclerosis such as myeloperoxidase (MPO) [8], coronary artery calcium (CAC) [9] and phosphorus [10]. Alkaline phosphatase (ALP) has enzyme catalytic function that induces the hydrolysis of organic pyrophosphate [11], and plays an important role in modulating inflammation process, mineral metabolism and vascular calcification [12]. Recently, some studies suggested a significant association between ALP and the pathogenesis of CAD [12,13]. Furthermore, ALP has been reported to predict the mortality, myocardial infarction or stent thrombosis in CAD patients following percutaneous coronary intervention (PCI) [11], which is a useful therapy to treat CAD that evidently improved the prognosis of CAD patients [14,15]. In addition, a low albumin (ALB) level has been also considered as a powerful biomarker to reflect the onset [16], progress and adverse outcomes [17,18] of CAD. Pu et al. reported that alkaline phosphatase-to-albumin ratio (AAR) was associated with the liver damage and poorer prognosis of patients with digestive system malignancy [19]. Moreover, several previous studies showed that there was a higher incidence of malignancy in patients with CAD [20–22]. However, it is unknown whether AAR is associated with adverse prognosis in CAD patients. To the best of our knowledge, there is no previous study investigating the relation between AAR and long-term adverse outcomes in post-PCI patients with CAD. Considering that ALP and ALB are involved in the onset, development and prognosis of CAD, it may be feasible to evaluate the potential value of AAR as a biomarker in predicting the adverse outcomes of CAD patients. Therefore, in our study, we aim to assess the relation between AAR and long-term adverse outcomes in CAD patients after undergoing PCI.

## Methods

### Study population and design

**Figure 1:**
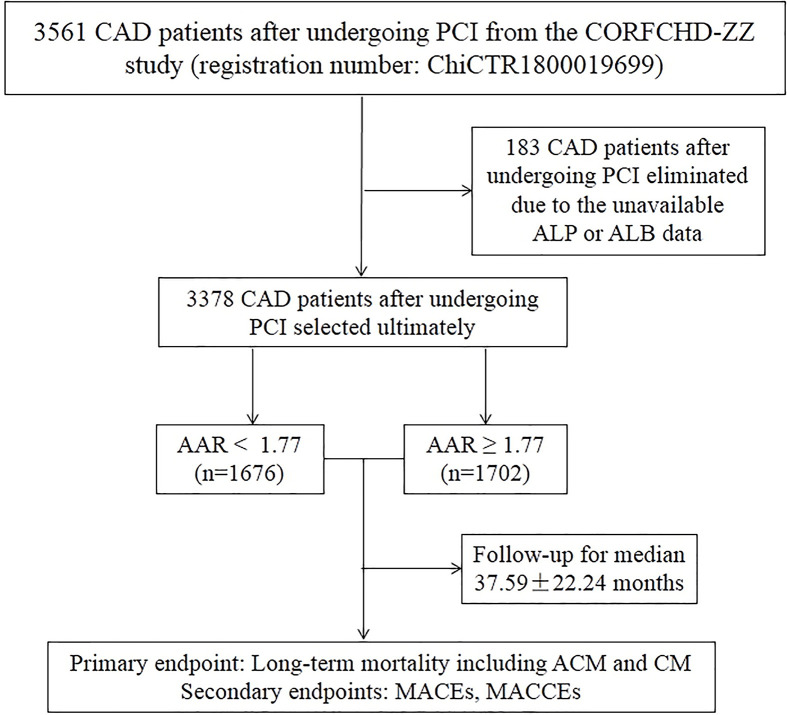
A flowchart of the study design Abbreviations: ACM, all-cause mortality; CM, cardiac mortality; MACE, major adverse cardiac event; MACCE, major adverse cardiac and cerebrovascular event.

In our study, all the patients were from the Clinical Outcomes and Risk Factors of Patients with Coronary Heart Disease after PCI (CORFCHD-ZZ) study, which was a large, retrospective cohort study including 3561 CAD patients following PCI from the First Affiliated Hospital of Zhengzhou University from January 2013 to December 2017 and their data were obtained from case records and follow-ups. The details of the CORFCHD-ZZ study could be browsed on http://www.chictr.org.cn (registration number: ChiCTR1800019699). A total of 3561 patients were initially enrolled; 183 patients were subsequently excluded due to unavailable baseline ALP or ALB data. Finally, there were 3378 eligible patients in our study. The inclusion criteria for eligibility in the current analysis were as follows: (1) patients aged at least 18 years; (2) at least one instance of coronary artery stenosis ≥ 50% confirmed by coronary angiography; (3) at least one clinical phenotype of coronary heart disease: stable angina or acute coronary syndrome and (4) an indispensable and objective check for evidence of myocardial ischemia: positive stress test, FFR < 0.80 or OCT or IVUS examination suggesting unstable plaque. Patients with the following baseline characteristics were excluded: (1) younger than 18 or older than 80; (2) severe valvular heart disease; (3) severe congenital heart disease; (4) hyperthyroidism, anemia or other high-powered heart disease; (5) pulmonary heart disease; (6) hypertrophic obstructive cardiomyopathy; (7) liver dysfunction (defined as alanine aminotransferase (ALT) or total bilirubin greater than 3-times the normal upper limit); (8) renal insufficiency (defined as serum creatinine (Cr) greater than 1.5-times the normal upper limit) or (9) conditions with a high-risk of bleeding, such as thrombocytopenia, blood diseases and other diseases. A flowchart of the study design was shown in Figure 1.

### Demographic, clinical and laboratory characteristics

All the data were collected from the case records of inpatients at the First Affiliated Hospital of Zhengzhou University including the demographic, clinical and laboratory data. We recorded gender, age, family history of CAD, medications, hypertension, diabetes, smoking, alcohol consumption, systolic blood pressure (SBP), diastolic blood pressure (DBP) and heart rate (HR) as the demographic and clinical data. The definitions of CAD, hypertension, diabetes, smoking and alcohol consumption were described previously [23]. In addition, we collected the laboratory data including plasma and biochemical parameters such as the levels of blood urea nitrogen (BUN), Cr, glucose (GLU), uric acid (UA), triglyceride (TG), total cholesterol (TC), high-density lipoprotein cholesterol (HDL-C), LDL-C, ALB, left ventricular ejection fraction (LVEF), pro-brain natriuretic peptide (Pro-BNP), ALT, aspartate aminotransferase (AST), γ-glutamyl transpeptidase (GGT) and ALP. All blood samples were collected through a standard venipuncture technique before performing coronary angiography after at least 12 h of fasting.

### End points and follow-up

The primary end point was long-term mortality, including all-cause mortality (ACM) and cardiac mortality (CM). The secondary end points were the composite of major adverse cardiac events (MACEs) defined as cardiac death, heart failure, bleeding events and re-admission, and the composite of major adverse cardiac and cerebrovascular events (MACCEs) defined as MACEs combined with stroke. All end points have been described previously [23].

The mean duration of follow-up time was 37.59 ± 22.24 months. All investigators underwent standard training on the following: (1) methods of follow-up: telephone interviews or office visits; (2) content of follow-up: complying with medical advice, the onset of end points and so on. The follow-up was conducted according to the above uniform criterion.

### Statistical analysis

All analyses were performed using SPSS version 22.0 (SPSS Inc., Chicago, Illinois, United States). Continuous variables were presented as the mean ± standard error and compared using *t* tests (for data complying with a normal distribution) or Mann–Whitney U-tests (for data complying with a non-normal distribution). Categorical variables were presented as frequencies and percentages and compared using the chi-square test. Receiver operating characteristic (ROC) curve was performed to determine the cut-off value of AAR (<1.77 and ≥1.77). The cumulative incidences of long-term outcomes were evaluated using the Kaplan–Meier method and compared using the log-rank test. Collinearity analysis was performed to evaluate whether there was collinearity among univariables with statistical differences before conducting multivariate analysis. Multivariate Cox proportional hazards regression models were conducted to evaluate the predictive performance of AAR to long-term outcomes. All *P*-values <0.05 were assumed to be significant.

## Results

### Baseline characteristics

In our study, the ROC curve showed that the cut-off value of AAR was 1.77, which divided a total of 3378 CAD patients after undergoing PCI into two groups: the lower group (AAR < 1.77, *n*=1676) and the higher group (AAR ≥ 1.77, *n*=1702). As shown in Table 1, we found that several variables were significantly different between the two groups, such as gender, alcohol consumption, age, DBP, BUN, UA, GLU, TC, HDL-C, LDL-C, ALB, LVEF, Pro-BNP, ALT, AST, GGT and ALP (all *P*<0.05).

**Table 1:** Baseline characteristics of patients

Variables	AAR < 1.77	AAR ≥ 1.77	χ^2^ or *t* or MWU	*P*-value
Family history, *n* (%)	327 (19.7)	308 (18.2)	1.148	0.284
Gender, male, *n* (%)	1212 (72.3)	1115 (65.5)	18.239	**<0.001**
Hypertension, *n* (%)	955 (57.0)	922 (54.2)	2.699	0.100
Diabetes, *n* (%)	403 (24.0)	390 (22.9)	0.601	0.438
Smoking, *n* (%)	526 (31.4)	502 (29.5)	1.424	0.233
Alcohol consumption, *n* (%)	298 (17.8)	251 (14.7)	5.708	**0.017**
Aspirin, *n* (%)	1676 (100)	1702 (100)		
Ticagrelor or Clopidogrel, *n* (%)	1676 (100)	1702 (100)		
β-blocker, *n* (%)	1184 (93.9)	1217 (93.0)	0.889	0.346
ACEI or ARB, *n* (%)	697 (41.6)	727 (42.7)	0.440	0.507
Statins, *n* (%)	1671 (99.7)	1692 (99.4)	1.598	0.206
Age, years	62.59 ± 10.87	63.94 ± 10.38	−3.695	**<0.001**
SBP, mm Hg	133.00 ± 17.11	132.80 ± 18.49	0.317	0.751
DBP, mm Hg	79.56 ± 11.12	78.75 ± 11.12	0.644	**0.035**
Heart rate, bpm	74.42 ± 22.73	74.77 ± 11.45	−0.567	0.571
BUN, mmol/l	5.52 ± 3.11	5.82 ± 4.94	−2.098	**0.036**
Cr, umol/l	71.87 ± 19.97	73.60 ± 45.69	−1.423	0.155
UA, mmol/l	301.97 ± 83.17	295.85 ± 88.33	2.063	**0.039**
GLU, mmol/l	5.46 ± 1.68	5.82 ± 2.54	−4.777	**<0.001**
TG, mmol/l	1.65 ± 1.15	1.68 ± 1.10	0.738	0.458
TC, mmol/l	3.84 ± 0.99	3.96 ± 1.05	−3.369	**0.001**
HDL-C, mmol/l	1.05 ± 0.27	1.03 ± 0.31	2.066	**0.039**
LDL-C, mmol/l	2.33 ± 0.82	2.46 ± 0.87	−4.200	**<0.001**
ALB, g/l	41.87 ± 4.30	39.77 ± 4.53	13.877	**<0.001**
LVEF, %	60.77 ± 6.95	59.81 ± 7.56	3.477	**0.001**
Pro-BNP, pg/ml	238.00 (101.25–594.50)	313.00 (139.00–823.15)	−6.002	**<0.001**
ALT, U/l	23.00 (15.00–38.00)	26.00 (17.00–45.00)	−5.578	**<0.001**
AST, U/l	21.00 (17.00–30.00)	22.00 (17.00–38.00)	−5.382	**<0.001**
GGT, U/l	23.00 (16.00–35.00)	29.00 (18.00–48.325)	−9.019	**<0.001**
ALP, U/l	61.00 (54.00–67.95)	86.00 (77.00–98.00)	−45.970	**<0.001**

The boldfaced *P*-values are statistically different. Abbreviations: ACEI, angiotensin-converting enzyme inhibitor; ARB, angiotensin receptor blocker; MWU, Mann–Whitney U-test.

However, there were no significant differences in the following variables between the two groups: family history; hypertension; diabetes; smoking; aspirin; ticagrelor or clopidogrel; β-blocker; angiotensin-converting enzyme inhibitor (ACEI) or angiotensin receptor blocker (ARB); Statins; SBP; HR; Cr and TG (all *P*≥0.05).

### Outcomes

**Figure 2:**
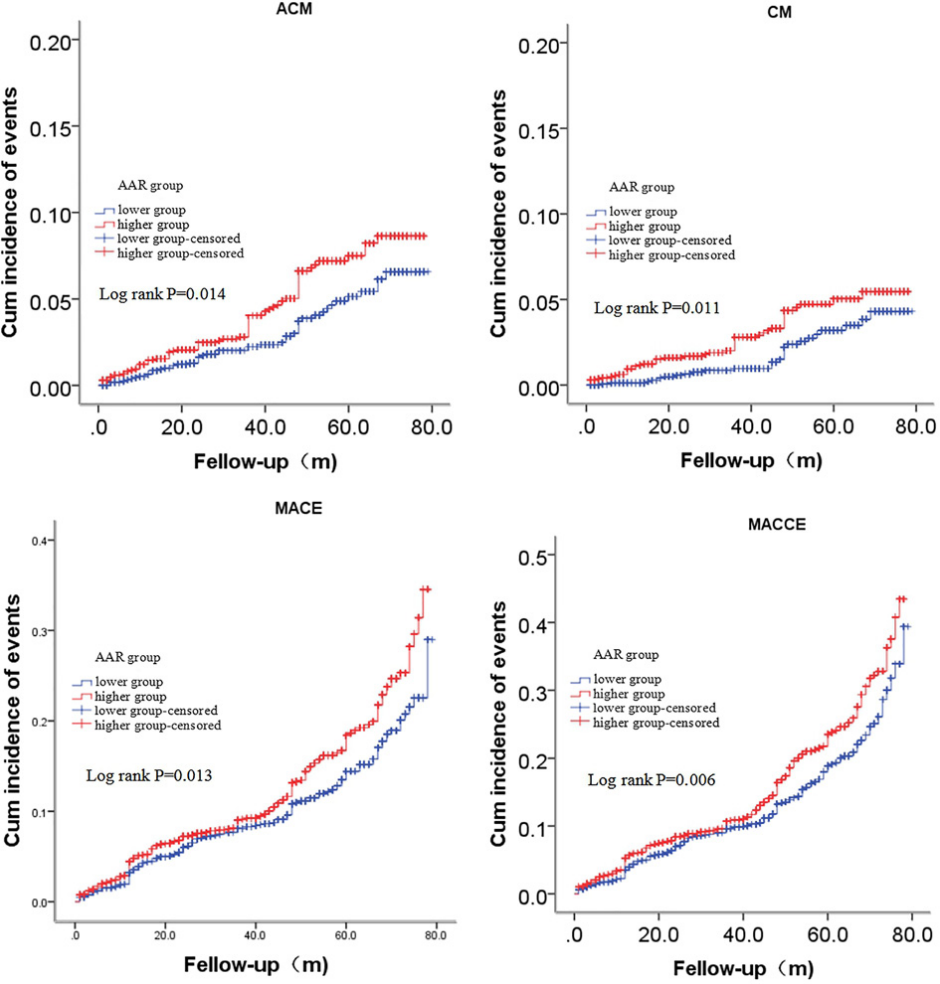
Cumulative Kaplan–Meier estimates of the time to the first adjudicated occurrence of ACM, CM, MACEs and MACCEs

As shown in Table 2, the incidence of ACM, CM, MACCEs and MACEs in lower AAR group were 3.0% (*n*=51), 1.7% (*n*=29), 10.2% (*n*=171) and 13.4% (*n*=225), respectively, and that in higher AAR group were 4.5% (*n*=76), 2.9% (*n*=50), 12.2% (*n*=208) and 15.8% (*n*=269). As shown in Figure 2, Kaplan–Meier analyses were performed to evaluate the cumulative incidences of long-term outcomes in both groups, and showed that an elevated AAR was significantly associated with long-term ACM (log-rank, *P*=0.014), CM (log-rank, *P=*0.011), MACEs (log-rank, *P=*0.013) and MACCEs (log-rank, *P=*0.006). Collinearity analysis was conducted to evaluate whether there was collinearity (VIF > 2 indicating obvious collinearity) among univariables with statistical differences before performing multivariate analysis; as shown in Table 3, the analysis showed that there was obvious collinearity between TC (VIF: 3.957) and LDL-C (VIF: 3.803). However, there was no collinearity among remaining univariables after excluding TC. And then, multivariate Cox proportional hazards regression models were conducted to evaluate whether there was a significant correlation between the AAR and long-term outcomes; the models were adjusted for confounders including gender, age, alcohol consumption, BUN, UA, HDL-C and LDL-C. Patients in the higher AAR group had an increased long-term ACM (adjusted HR = 1.488 [1.031–2.149], *P*=0.034), CM (adjusted HR = 1.837 [1.141–2.959], *P=*0.012), MACEs (adjusted HR = 1.257 [1.018–1.551], *P*=0.033) and MACCEs (adjusted HR = 1.237 [1.029–1.486], *P*=0.024) incidence, compared with patients in the lower AAR group after being adjusted for the abovementioned confounders (Table 4). Therefore, the increased AAR was an independent predictor of long-term adverse outcomes in CAD patients after undergoing PCI. In addition, all adjusted confounders of long-term ACM, CM, MACEs and MACCEs were shown in Supplementary Tables S1–S4.

**Table 2:** Outcomes comparison between both groups on log-rank test

Outcomes	AAR < 1.77	AAR ≥ 1.77	χ^2^	*P*-value
ACM, *n* (%)	51 (3.0)	76 (4.5)	6.038	**0.014**
CM, *n* (%)	29 (1.7)	50 (2.9)	6.502	**0.011**
MACEs, *n* (%)	171 (10.2)	208 (12.2)	6.138	**0.013**
MACCEs, *n* (%)	225 (13.4)	269 (15.8)	7.420	**0.006**

The boldfaced *P*-values are statistically different.

**Table 3:** Collinearity analysis for confounders

Variables	B	SE	β	T	*P*-value	TOL	VIF
Constant	−0.148	0.030		−4.977	<0.001		
Gender	−0.010	0.008	−0.024	−1.249	0.212	0.795	1.258
Age	0.003	0.000	0.154	8.620	<0.001	0.938	1.067
Alcohol consumption	0.009	0.009	0.018	0.986	0.324	0.908	1.101
BUN	0.003	0.001	0.062	3.541	<0.001	0.971	1.030
UA	6.469E^−5^	0.000	0.029	1.574	0.116	0.891	1.122
TC	0.013	0.006	0.068	1.960	0.050	0.253	**3.957**
HDL-C	−0.026	0.012	−0.040	−2.149	0.032	0.866	1.155
LDL-C	−0.016	0.008	−0.071	−2.107	0.035	0.263	**3.803**

The boldfaced VIF indicates that there is obvious collinearity between TC and LDL-C.

**Table 4:** Incidence of outcomes on multivariate Cox proportional hazards regression models

Outcomes	HR (95% CI)	*P*-value	Adjusted HR (95% CI)^1^	*P*-value
ACM	1.554 (1.090–2.216)	**0.015**	1.488 (1.031–2.149)	**0.034**
CM	1.797 (1.137–2.841)	**0.012**	1.837 (1.141–2.959)	**0.012**
MACEs	1.290 (1.053–1.579)	**0.014**	1.257 (1.018–1.551)	**0.033**
MACCEs	1.277 (1.070–1.525)	**0.007**	1.237 (1.029–1.486)	**0.024**

The boldfaced *P*-values are statistically different.

1 Adjusted for gender, age, alcohol consumption, BUN, UA, HDL-C and LDL-C.

## Discussion

To the best of our knowledge, this is the first study evaluating the prognostic value of AAR to long-term outcomes in CAD patients. In our study, we found that an increased AAR was an independent predictor of long-term adverse outcomes in CAD patients following PCI after being adjusted for several confounders including gender, age, alcohol consumption, BUN, UA, HDL-C and LDL-C. Moreover, our study had a large sample (3378 enrolled patents) which improved the statistical power and made the results more credible.

Since CAD has been a major cause of mortality [1], a large number of biomarkers, such as hsCRP [2], LDL-C [5], d-dimer [6], CAC [9] and ALB [16–18], were investigated whether there was a significant relation with CAD, and the results suggested that these typical biomarkers had powerful predictive performance to poor prognosis of CAD. Furthermore, recent studies demonstrated that several emerging novel biomarkers were considered as independent predictors of long-term outcomes in post-PCI patient with CAD such as ALP [11,13], plasma mannose [24], liver miRNAs [25], cysteine-rich angiogenic inducer 61 (Cyr61) [26] and Amyloid-β (1-40) [27]. In a retrospective study, Pu et al. found that AAR was associated with the liver damage and poorer prognosis of patients with digestive system malignancy and an elevated AAR reflected the increase in tumor site and several serum biochemical indexes levels [19]. With the rapid development of tumor detection and antitumor therapy, there was a higher prevalence of malignancy in patients with CAD [20–22], such as the BleeMACS study [20] subanalysis demonstrated a non-negligible prevalence with a higher incidence of death, re-infarction and bleeding in a CAD subpopulation with malignancy compared with the overall CAD population. Similarly, CAD patients with malignancy were at higher risk of in-hospital and long-term mortality as compared with non-malignant patients in another previous study [22] which showed the related mechanisms were endothelial dysfunction, increased expression of pro-inflammatory cytokines, oxidative stress and platelet activity. Moreover, AAR was also demonstrated as a novel inflammatory marker of poor prognosis in patients with malignancy [19]. In addition, both ALP and ALB were involved in the onset, development and prognosis of CAD. Based on these previous findings, we logically hypothesized that AAR would be an effective predictor for adverse outcomes in CAD patients who undergo PCI.

ALP could be activated by oxidative stress, and its increase was also associated with oxidative stress [28]. Oxidative stress, a risk factor for CAD, powerfully reflected the initiation of atherosclerosis, and NADPH oxidases were able to produce reactive oxygen species [29] which was harmful to DNA, lipids and proteins [19]. Similarly, in a recent review enrolling more than 1000 studies [30], Tejero et al. found that dysregulated production of reactive oxygen species (ROS) or reactive nitrogen species (RNS), such as NO, lead to oxidative stress and in turn induced the onset and development of CAD, and they interpreted particularly the related mechanisms of lots of signaling molecules such as Tyr^657^, heme-depleted sGC and NOXs. It is well-known that several mechanisms are involved in the pathogenesis of CAD such as inflammatory response [3,7], metabolic disturbance [4,5] and atherosclerosis [9]. Furthermore, an elevated ALP level played an important role in contributing to inflammation process, inducing abnormal mineral metabolism and accelerating the initiation of atherosclerosis, and then leading to the onset of CAD [12]. In addition, ALP was independently associated with the adverse outcomes in CAD patients from Iran, such as stroke and ACM [31]. A similar study also showed that ALP was a powerful predictor of mortality, myocardial infarction or stent thrombosis in CAD patients following PCI [11]. On the contrary, ALB was considered as a protective factor for cardiac function [32] and its decrease was positively associated with the incidence and long-term mortality of CAD [16,33]. A low ALB level could attenuate fibrinolysis, decrease antioxidant capacity, disrupt endothelial functions, activate inflammatory process, increase blood viscosity and the risk of atherothrombosis, leading to adverse cardiovascular events [34]. Furthermore, a recent study reported that autophagy may be considered as a related mechanism on how a low ALB level induced the onset of cardiovascular events in CAD patients: a reduced serum ALB level was able to induce autophagy; excessive autophagy contributed to the death of pancreatic β-cells leading to impaired glucose tolerance, and it could impair myocardial cells leading to reduced cardiac function; therefore, a low ALB level increased the incidence of cardiovascular events [35]. More recently, Wada et al. demonstrated that a low ALB level was an independent predictor of long-term mortality in CAD patients without chronic kidney disease after undergoing PCI [36].

Considering that increased ALP and reduced ALB levels in serum were significantly associated with the pathogenesis of CAD and contributed to the initiation, progress and prognosis of CAD, plus our findings from a cohort study with a large sample, thus we thought that AAR may be a reasonable and feasible biomarker to predict long-term adverse outcomes in post-PCI patients with CAD.

## Study limitations

First, there were some unavailable baseline ALP and ALB data from a few patients in our study, and the removal of these patients contributed to the reduction in study sample size. Second, we did not take atrial fibrillation into consideration in the exclusion criteria. Third, it was not considered whether fracture healing, some bone diseases and corticosteroids had some influences on AAR. Fourth, we only collected the baseline data, so it was unknown whether dynamic change of these variables affected the end points. Fifth, our study was a single, retrospective study and only evaluate the relation between AAR and long-term adverse outcomes in post-PCI patients with CAD from China, thus the findings still need to be further demonstrated in other populations.

## Conclusions

An elevated AAR is a novel independent predictor of long-term adverse outcomes in CAD patients after undergoing PCI, such as mortality, MACEs and MACCEs, and it is worth utilizing in clinical practice.

## Supplementary Material

Supplementary Tables S1-S4

## Data Availability

The data will not be shared, because the identified participants information is included in the data.

## Abbreviations

AAR: alkaline phosphatase-to-albumin ratio
ACM: all-cause mortality
ALB: albumin
ALP: alkaline phosphatase
ALT: alanine aminotransferase
AST: aspartate aminotransferase
BUN: blood urea nitrogen
CAC: coronary artery calcium
CAD: coronary artery disease
CM: cardiac mortality
CORFCHD-ZZ: Clinical Outcomes and Risk Factors of Patients with Coronary Heart Disease after PCI
Cr: creatinine
DBP: diastolic blood pressure
FFR: fraction flow reserve
GGT: γ-glutamyl transpeptidase
GLU: glucose
HDL-C: high-density lipoprotein cholesterol
hsCRP: high-sensitivity C-reactive protein
IVUS: intravascular ultrasound
LDL-C: low-density lipoprotein cholesterol
LVEF: left ventricular ejection fraction
MACCE: major adverse cardiac and cerebrovascular event
MACE: major adverse cardiac event
MPO: myeloperoxidase
NOX: NADPH oxidase
OCT: optical coherence tomography
PCI: percutaneous coronary intervention
Pro-BNP: pro-brain natriuretic peptide
ROC: receiver operating characteristic
SBP: systolic blood pressure
sGC: soluble guanylyl cyclase
TC: total cholesterol
TG: triglyceride
UA: uric acid
VIF: variance inflation factor
