# Supplementary material for: Alkaline phosphatase-to-albumin ratio as a novel predictor of long-term adverse outcomes in coronary artery disease patients who underwent PCI
Source: Biosci Rep. 2021 Jun 29;41(7):BSR20203904. doi: 10.1042/BSR20203904 (PMC8243337; doi:10.1042/BSR20203904)
Supplement: Supplementary Tables S1-S4 [file BSR-2020-3904_supp.pdf]

Table S1: Cox regression analysis results for long-term ACM

| Variables | B | SE | Wald | *P*-Value | HR | 95%CI |
|---|---|---|---|---|---|---|
| Gender | -0.255 | 0.227 | 1.258 | 0.262 | 0.775 | 0.496-1.210 |
| Age | 0.072 | 0.010 | 55.258 | **<0.001** | 1.075 | 1.054-1.095 |
| Alcohol consumption | 0.258 | 0.244 | 1.118 | 0.290 | 1.295 | 0.802-2.090 |
| BUN | 0.048 | 0.012 | 15.732 | **<0.001** | 1.049 | 1.024-1.074 |
| UA | 0.001 | 0.001 | 1.703 | 0.192 | 1.001 | 0.999-1.003 |
| HDL-C | -0.529 | 0.368 | 2.067 | 0.150 | 0.589 | 0.286-1.212 |
| LDL-C | -0.057 | 0.111 | 0.267 | 0.606 | 0.944 | 0.759-1.174 |
| AAR | 0.398 | 0.187 | 4.503 | **0.034** | 1.488 | 1.031-2.149 |

Abbreviation: ACM, all-cause mortality; BUN, blood urea nitrogen; UA, uric acid; HDL-C, high-density lipoprotein cholesterol; LDL-C, low-density lipoprotein cholesterol; AAR, alkaline phosphatase to albumin ratio. Note: The boldfaced *P*-Values are statistically different.

Table S2: Cox regression analysis results for long-term CM

| Variables | B | SE | Wald | *P*-Value | HR | 95%CI |
|---|---|---|---|---|---|---|
| Gender | -0.681 | 0.318 | 4.582 | **0.032** | 0.506 | 0.271-0.944 |
| Age | 0.068 | 0.012 | 31.764 | **<0.001** | 1.070 | 1.045-1.095 |
| Alcohol consumption | 0.011 | 0.316 | 0.001 | 0.971 | 1.011 | 0.544-1.879 |
| BUN | 0.049 | 0.013 | 13.979 | **<0.001** | 1.050 | 1.024-1.078 |
| UA | 0.002 | 0.001 | 3.610 | 0.057 | 1.002 | 1.000-1.005 |
| HDL-C | -0.911 | 0.487 | 3.500 | 0.061 | 0.402 | 0.155-1.044 |
| LDL-C | -0.044 | 0.142 | 0.097 | 0.755 | 0.957 | 0.725-1.263 |
| AAR | 0.608 | 0.243 | 6.258 | **0.012** | 1.837 | 1.141-2.959 |

Abbreviation: CM, cardiac mortality; BUN, blood urea nitrogen; UA, uric acid; HDL-C, high-density lipoprotein cholesterol; LDL-C, low-density lipoprotein cholesterol; AAR, alkaline phosphatase to albumin ratio. Note: The boldfaced *P*-Values are statistically different.

Table S3: Cox regression analysis results for MACEs

| Variables | B | SE | Wald | *P*-Value | HR | 95%CI |
|---|---|---|---|---|---|---|
| Gender | -0.099 | 0.132 | 0.565 | 0.452 | 0.906 | 0.700-1.172 |
| Age | 0.007 | 0.005 | 1.652 | 0.199 | 1.007 | 0.996-1.017 |
| Alcohol consumption | -0.016 | 0.146 | 0.012 | 0.912 | 0.984 | 0.738-1.311 |
| BUN | 0.019 | 0.010 | 3.764 | 0.052 | 1.019 | 1.000-1.039 |
| UA | 0.000 | 0.001 | 0.369 | 0.544 | 1.000 | 0.999-1.002 |
| HDL-C | -0.368 | 0.215 | 2.940 | 0.086 | 0.692 | 0.454-1.054 |
| LDL-C | 0.097 | 0.062 | 2.451 | 0.117 | 1.102 | 0.976-1.244 |
| AAR | 0.228 | 0.107 | 4.534 | **0.033** | 1.257 | 1.018-1.551 |

Abbreviation: MACEs, major adverse cardiovascular events; BUN, blood urea nitrogen; UA, uric acid; HDL-C, high-density lipoprotein cholesterol; LDL-C, low-density lipoprotein cholesterol; AAR, alkaline phosphatase to albumin ratio. Note: The boldfaced *P*-Values are statistically different.

Table S4: Cox regression analysis results for MACCEs

| Variables | B | SE | Wald | *P*-Value | HR | 95%CI |
| --- | --- | --- | --- | --- | --- | --- |
| Gender | -0.058 | 0.113 | 0.264 | 0.608 | 0.943 | 0.756-1.178 |
| Age | 0.010 | 0.005 | 5.075 | **0.024** | 1.010 | 1.001-1.020 |
| Alcohol consumption | -0.098 | 0.133 | 0.540 | 0.463 | 0.907 | 0.699-1.177 |
| BUN | 0.018 | 0.009 | 4.234 | **0.040** | 1.018 | 1.001-1.036 |
| UA | 0.000 | 0.001 | 0.604 | 0.437 | 1.000 | 0.999-1.002 |
| HDL-C | -0.243 | 0.184 | 1.759 | 0.185 | 0.784 | 0.547-1.123 |
| LDL-C | 0.097 | 0.054 | 3.204 | 0.073 | 1.102 | 0.991-1.225 |
| AAR | 0.212 | 0.094 | 5.126 | **0.024** | 1.237 | 1.029-1.486 |

Abbreviation: MACCEs, major adverse cardiovascular and cerebrovascular events; BUN, blood urea nitrogen; UA, uric acid; HDL-C, high-density lipoprotein cholesterol; LDL-C, low-density lipoprotein cholesterol; AAR, alkaline phosphatase to albumin ratio. Note: The boldfaced *P*-Values are statistically different.
